# The rights and wrongs of blood-brain barrier permeability studies: a walk through 100 years of history

**DOI:** 10.3389/fnins.2014.00404

**Published:** 2014-12-16

**Authors:** Norman R. Saunders, Jean-Jacques Dreifuss, Katarzyna M. Dziegielewska, Pia A. Johansson, Mark D. Habgood, Kjeld Møllgård, Hans-Christian Bauer

**Affiliations:** ^1^Department of Pharmacology and Therapeutics, University of MelbourneParkville, VIC, Australia; ^2^Department of Neuroscience, University of GenevaGeneva, Switzerland; ^3^Institute for Stem Cell Research, Helmholtz Center MunichMunich, Germany; ^4^Department of Cellular and Molecular Medicine, University of CopenhagenCopenhagen, Denmark; ^5^Institute of Tendon and Bone Regeneration, Paracelsus Medical UniversitySalzburg, Austria; ^6^Spinal Cord Injury and Tissue Regeneration Center, Paracelsus Medical UniversitySalzburg, Austria

**Keywords:** blood-brain barrier, blood-cerebrospinal fluid barrier, embryo, fetus, newborn, permeability, tight junctions, transporters

## Abstract

Careful examination of relevant literature shows that many of the most cherished concepts of the blood-brain barrier are incorrect. These include an almost mythological belief in its immaturity that is unfortunately often equated with absence or at least leakiness in the embryo and fetus. The original concept of a blood-brain barrier is often attributed to Ehrlich; however, he did not accept that permeability of cerebral vessels was different from other organs. Goldmann is often credited with the first experiments showing dye (trypan blue) exclusion from the brain when injected systemically, but not when injected directly into it. Rarely cited are earlier experiments of Bouffard and of Franke who showed methylene blue and trypan red stained all tissues except the brain. The term “blood-brain barrier” “Blut-Hirnschranke” is often attributed to Lewandowsky, but it does not appear in his papers. The first person to use this term seems to be Stern in the early 1920s. Studies in embryos by Stern and colleagues, Weed and Wislocki showed results similar to those in adult animals. These were well-conducted experiments made a century ago, thus the persistence of a belief in barrier immaturity is puzzling. As discussed in this review, evidence for this belief, is of poor experimental quality, often misinterpreted and often not properly cited. The functional state of blood-brain barrier mechanisms in the fetus is an important biological phenomenon with implications for normal brain development. It is also important for clinicians to have proper evidence on which to advise pregnant women who may need to take medications for serious medical conditions. Beliefs in immaturity of the blood-brain barrier have held the field back for decades. Their history illustrates the importance of taking account of all the evidence and assessing its quality, rather than selecting papers that supports a preconceived notion or intuitive belief. This review attempts to right the wrongs. Based on careful translation of original papers, some published a century ago, as well as providing discussion of studies claiming to show barrier immaturity, we hope that readers will have evidence on which to base their own conclusions.

## Introduction

**Figure 1:**
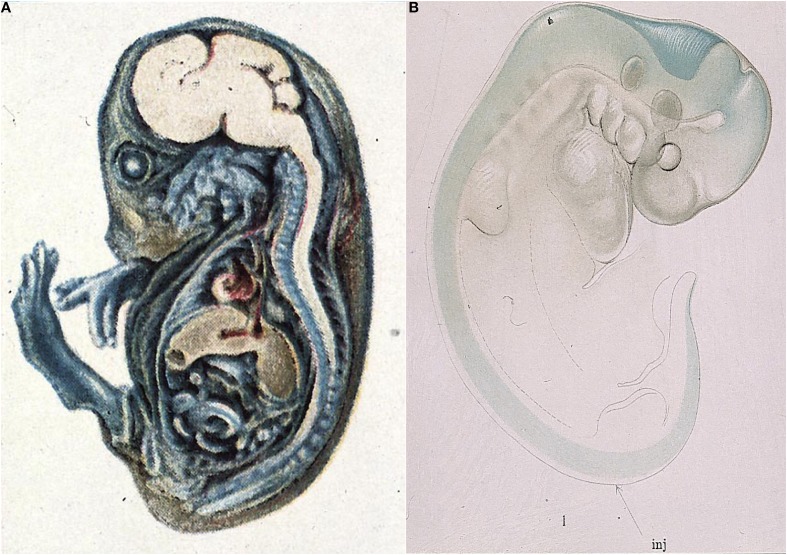
**Early demonstration of blood-brain barrier phenomenon in developing brain. (A)** Mid gestation guinea pig embryo injected with trypan blue (Wislocki, 1920). Note lack of staining of brain and spinal cord as previously described in adult animals injected with trypan blue (Goldmann, 1909), methylene blue (Bouffard, 1906) and trypan red (Franke, 1905). **(B)** Very early pig embryo (9 mm, E19) injected with isotonic sodium ferrocyanide into spinal canal (arrow) treated with acid (Prussian blue reaction). Note that blue reaction product is confined to CSF, with a small amount of diffusion into brain stem and mid brain tissue, but no staining of somatic tissue. This indicates that the CNS is already a closed compartment separate from the rest of the embryo. From Weed (1917b).

The very first studies of barrier properties in embryos clearly demonstrated the intactness of the interfaces between the blood, brain and cerebrospinal fluid^1^. Thus Wislocki (1920) injected a guinea pig embryo with trypan blue (Figure 1A), the same dye, which was first used by Goldmann (1909; 2013 see below). Wislocki (1920) observed that in the embryo, as in adult animals, the dye stained almost all of the tissues of the body, with the notable exception of much of the brain.

Even earlier than this Weed (1917a,b) injected an approximately isotonic solution of potassium ferrocyanide, a small molecule about the same size as sucrose, into the neural tube of E19 and older pig embryos. Weed's results showed that in an E19 pig embryo the potassium ferrocyanide (demonstrated by the Prussian blue reaction) remained entirely within the fluid filling the neural tube (Figure 1B). This suggests that even this early in development, shortly after neural tube closure, the central nervous system (CNS) is separate from the rest of the embryo.

Similar results were obtained in chick embryos by Cohen and Davies (1938). The experiments of Wislocki, Weed and Cohen and Davies are the developmental equivalent of what are sometimes described as Goldmann's 1st and 2nd experiments, in which he reported iv injection (1909) and intrathecal injection (1913) of trypan blue in adult animals of various species. The evidence from the experiments of Weed, Wislocki, Chen and Davies in developing animals for the existence of a barrier between the blood and brain and blood and CSF was as convincing as Goldmann's experiments in adult animals. Yet at some point a belief, which has persisted for nearly 100 years, arose that in embryos, fetuses and even in the newborn and older, the blood-brain barrier is absent, leaky or immature (e.g., Statz and Felgenhauer, 1983; Adinolfi, 1985; Davies and Rudd, 1994; Rodier, 1994, 1995, 2004; Ahmed et al., 1996; ATSDR, 1999, 2007, 2014; Järup, 2003; Costa et al., 2004; Watson et al., 2006; Mhanna et al., 2008; Baldrick, 2010; Ehman, 2010; Srinivasan et al., 2012). These and other authors appear to equate immaturity with “leakiness.” The developing brain in all respects is by definition yet to reach a mature adult state and therefore can rightly be described as immature. But this does not tell us whether its brain barriers are “leaky.” The real question is their functional status, which seems to us to be more a matter of the relation between barrier functions (which are much more than the presence or absence of passive permeability) that are present at any particular age and stage of brain development. We wondered why such a dogma continues to be accepted in view of overwhelming experimental evidence to the contrary gathered over many decades, for as pointed out by Dobbing (1961): “There has never been a shred of satisfactory evidence in favor of this proposition.”

The general explanation seems to be a philosophical rather than an experimentally based one. There seems to have been an underlying assumption, often not clearly enunciated, that because the embryonic and newborn brains are developing, adult barrier mechanisms would necessarily be immature or absent. This was most explicitly stated in “The brain and its environment” by the English physiologist Barcroft (1938): “There is no reason why the brain of the embryo should require an environment of very great chemical constancy. It will of course require a certain minimum of the various materials necessary for growth, but otherwise on first principles we might suppose that the good things of life may exist in and may vary in fetal blood to an extent much greater than in the maternal.” However, this is a teleological argument, implying need/requirement or in this case, lack of need/requirement. One could as well argue that a complex organ like the brain would need (require) a specialized environment in which to develop rather than the general “soup” of the embryo as a whole. However, neither argument has any intellectual merit as they are based on opinion rather than experimental evidence. More recently, and in the face of much evidence to the contrary since Barcroft's time, Zheng (2002) has put forward a similar teleological argument: “During early development, the rapid growth of the cerebral cortex perhaps necessities a “leaky” structure of the BBB to accommodate the high demand of blood-borne nutrients for brain growth.” However, as we discuss below, the greater entry of some amino acids into the developing brain is a reflection of much higher expression of their transporters in brain endothelial and choroid plexus epithelial cells. This higher expression, which indicates a developmental specialization, is likely to be a reflection of the importance of amino acids for developing brain rather than a manifestation of a non-specific leak.

## Concept of the blood-brain barrier

Before dealing with experimental evidence of brain barriers during development we need to consider the concept of the adult blood-brain barrier itself. This is often attributed to Ehrlich on the basis of his experiments with dyes (e.g., Gröntoft, 1954; Bakay, 1956; Dempsey and Wislocki, 1955; Lee, 1971; Davson et al., 1987; Risau and Seulberger, 1990; Risau and Wolburg, 1990; Hawkins and Davis, 2005; Nag, 2011). However, as pointed out by Bradbury (1995); Ehrlich (1885, 1906) himself never subscribed to the view that the lack of staining of the brain by parenterally administered dyes could be ascribed to some special property of the cerebral endothelial cells. This idea was proposed by Biedl and Kraus (1898); Roux and Borrel (1898), and Lewandowsky (1900) based on comparison of intrathecal and parenteral injections of materials with neurotoxic effects, such as bile salts, after injection by the former route. This was specifically rejected by Ehrlich: “I am unable to accept that the vascular endothelium, as such, exercises different functions in different organs, so that, for example a liver capillary is permeable for certain substances that will not pass through other capillaries” (Ehrlich, 1906). This seems a rather curious conclusion given the description of some of his earlier dye studies (Ehrlich, 1885). In his extensive MD thesis (“Habilitationsschrift”) Ehrlich was primarily using dyes as a means of determining the oxygen usage in different organs by their color change (low oxygen or reduction resulting in a colorless state of the dye and on oxygenation resulted in the reverse). He used mainly Alizarin Blue-S (a compound of Alizarin blue and sodium bisulfite) and Indophenol white (which became blue when exposed to oxygen), injected usually subcutaneously into animals of various species. Both of these dyes stained the brain blue, exclusively in gray matter. Under conditions of reduced oxygen (as post mortem) the dyes became colorless. Ehrlich also used dyes treated with acids or heavy metals to give larger particle sizes-alizarin-sodium sulfite-alum and Coerulin-S. These did not stain the brain following subcutaneous injection. To explain this Ehrlich proposed, that “the brain was the most “finely porous” of the organs” he studied. Ehrlich does not say if these “pores” were in blood vessel walls or some other interface between blood and brain tissue. Later Ehrlich (1906) reported on a much larger number of dyes and concluded that acid dyes (with the exception of alizirin) did not stain the gray matter of the brain whereas many basic dyes did. He suggested that dye experiments could be explained by lack of affinity of brain tissue for the dyes rather than to a special property of brain blood vessels. So perhaps Ehrlich could best be described as the reluctant discoverer of the blood-brain barrier phenomenon, but not as the originator of the term “blood-brain barrier.”

Many authors, including Spatz (1934), Broman (1941), Davson (1967), Davson et al. (1987), Hawkins and Davis (2005), Engelhardt (2003, 2006), Ribatti et al. (2006), Saunders et al. (2012) have attributed the term “Blut-Hirnschranke” (blood-brain barrier) to Lewandowsky's (1900) publication.

Lewandowsky (1900) used strychnine and sodium ferrocyanide, in much higher doses than the potassium ferrocyanide used by Weed (1917b), who stated that in his experiments it was not toxic. Lewandowsky demonstrated in a variety of animal species that the toxins were effective in much lower doses when injected into the brain than parenterally. He also attributed the lesser penetration of strychnine and sodium ferrocyanide into the brain following subcutaneous injection compared to subarachnoid injection to a difference in the properties of the cerebral vessels compared to other tissues: “… die Hypothese dass die Cappillarwand den Uebertritt bestimmter Stoffe, wie des Natriumferrocyanats verhindert, oder mit anderen Worten, dass seine Affinitat der Capillarzellen des Centralnervensystems nothwendig ist um bestimmte Stoffe an die Nervenzellen hindurchgelangen zu lassen.”: *the hypothesis that the capillary wall blocks the transfer of certain molecules like sodium ferrocyanide or in other words, that an affinity of capillary cells of the CNS is necessary in order to allow the transfer of certain molecules to nerve cells*. A close reading of Lewandowsky's original paper reveals that the term “Blut-Hirnschranke” was not actually used, although as indicated by the above quotation, Lewandowsky clearly proposed that cerebral capillaries have specific restrictive properties with respect to some compounds.

The first use of the term “barrier” appears to be by Stern and Gautier (1918a,b), the latter paper is entitled “La barrière qui s'oppose au passage dans le liquide céphalo-rachidien de substances circulant dans le sang présente des différences notables suivant les espèces animales”: *The barrier which opposes the movement into the CSF of substances in the blood shows notable differences in different animal species* (Stern and Gautier, 1918b). Stern and colleagues subsequently performed detailed studies of penetration of a wide range of molecules from blood into cerebrospinal fluid (Stern and Gautier, 1921) into brain (Stern and Gautier, 1922) and within the subarachnoid and ventricular CSF (Stern and Gautier, 1923) of adult animals. It was Stern who first described the importance of the interstitial fluid environment of the brain for its normal function, very much in the tradition of Claude Bernard's *milieu interne* (1865).

Malamud et al. (1928), Franceschini (1929) and Joó (1987) appear to be among only a few authors to have acknowledged that Stern was probably the first to coin the term blood-brain barrier (“barrière hémato-encéphalique,” Stern and Gautier, 1921). Stern and Gautier were careful to point out that this term did not prejudge either the anatomical nature or functional mechanisms involved. The term “blood-brain barrier” has withstood the test of time, notwithstanding that to people outside the field it is misleading in that it implies an absolute impediment to entry into the brain and does not make it explicitly clear that exchange mechanism are involved—exactly as supposed by Stern when she proposed the term. However, Stern's important contributions to the field of blood-brain barrier biology have either been largely ignored or belittled (see below).

In addition to Lewandowsky's 1900 experiments, Biedl and Kraus (1898) injected bile and bile acids either systemically or directly into the brain. Only in the case of the latter did they see any toxic effects. They ascribed the ineffectiveness of systemically injected bile salts to “the relative impermeability of the capillary walls,” although they did not use the term “barrier” to describe this effect. Roux and Borrel (1898) reported on the comparison of subcutaneous and subdural injections of tetanus toxin with the finding that much less toxin is required to provoke central manifestations of tetanus, but only in rabbits; other species were unaffected. Their explanation for the difference was “sans doute, à ce que beaucoup du poison introduit n'arrive pas aux cellules nerveuses et est detruit quelque part dans l'organisme.”: without doubt little of the toxin reaches nervous tissue and is destroyed elsewhere in the organism. However, they did not comment on the mechanism that prevented toxin from reaching the brain.

**Figure 2:**
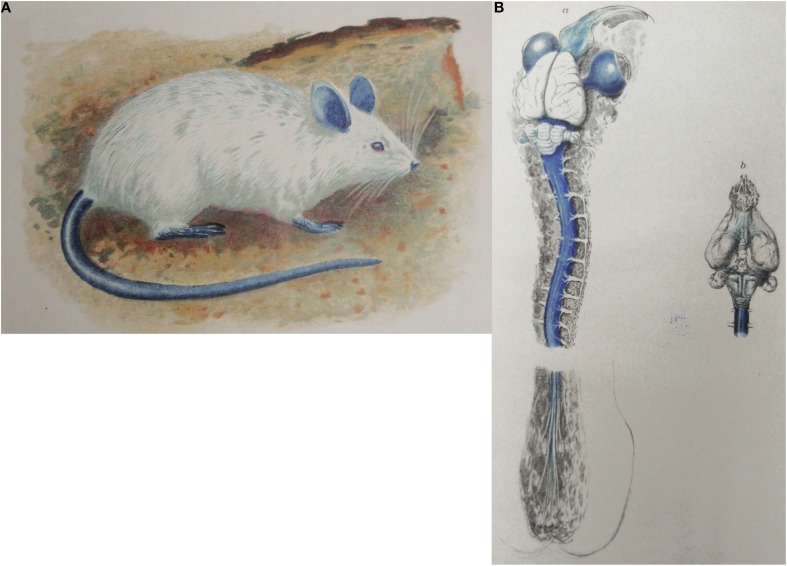
**Goldmann's trypan blue injection experiments. (A)** Adult rat following systemic injection of trypan blue solution (Goldmann's “1st experiment”). From Goldmann (1909). **(B)** Brain and spinal cord of adult rat following lumbar subarachnoid injection of 0.5 ml, 0.5% trypan blue (Goldmann's “2nd experiment”). From Goldmann (1913).

Amongst the most cited papers for the concept of the blood-brain barrier are those of Goldmann (1909, 1913). He compared distribution of the dye trypan blue following parenteral or intrathecal injection. These are sometimes referred to as Goldmann's first and second experiments (Davson, 1976; Bradbury, 1979). Goldmann found that parenterally injected dye did not stain the brain (Goldmann, 1909), whereas it did when injected into the subarachnoid space, (Goldmann, 1913) as illustrated in Figure 2. Goldmann is usually given the credit for being the first to use a dye to demonstrate that some dyes administered parenterally do not enter the brain. Goldmann himself acknowledges earlier dye studies of Bouffard (1906) but incorrectly states that Bouffard used trypan blue. Bouffard makes an unreferenced mention of even earlier studies with trypan red and Congo red and describes his own studies with methylene blue. Bouffard (1906) injected this dye by various routes (subcutaneous, intravenous, intramuscular or intracardiac) into mice, guinea pigs and rabbits in single or multiple doses of increasing size. He reported unequivocally that there was no staining of the nervous tissue: “SYSTEME NERVEUX.—Aucune trace de couleur, ni dans les cellules nerveuses, ni dans les éléments névrogliques.” Bouffard did not report any toxic effects of methylene blue and did not seem to suggest any mechanism to explain the lack of staining of the nervous system. Bouffard (1906) mentioned, but did not reference, an even earlier study by a student of Ehrlich's, Franke, who used trypan red. According to Bouffard, Franke also showed that the dye did not enter the brain although it did enter most other tissues and organs. This citation appears to have been taken from Franke's (1905) thesis, in which Franke stated “Das Trypanrot gehört zu den polytropen Farbstoffen im Sinne EHRLICHS; es wird entsprechend den Beobachtungen, die bei anderen saueren Farbstoffen gemacht worden sind, von allen Organen mit Ausnahme des Nervensystems, aufgenommen”: *Trypan red belongs to the polytropic dyes according to Ehrlich; it is taken up,- as observed with other acidic dyes, by all organs except the nervous system*. None of these authors seems to have offered any explanation of the lack of staining of the brain by the different dyes; indeed Goldmann (1913) appears to have been surprised by this observation: “Es ist mir rätselhaft dass grosse Farbstoffmengen, welche bei der intravenösen Applikation in den Gehirngefässen zirkulieren, selbst die feinste Kapillarwand nicht zu durchdringen vermögen”: *I fail to understand why high concentrations of dye circulating in the blood vessels after intravenous injection are unable to penetrate even the walls of the finest capillaries* (Goldmann, 1913, p. 45).

Goldmann (1913) did however, remark on the strong staining of the choroid plexus with trypan blue and suggested this represented the site at which dyes were prevented from entering the brain. Goldmann (1909, 1913) reported toxic effects with injected trypan blue and commented that the toxic effects that occurred following subarachnoid injections of the dye were made with amounts of trypan blue that were much less than when injected intravenously, particularly in rabbits. Because the choroid plexus stained very strongly with the dye, Goldmann suggested that it was primarily the choroid plexus that provided a protective mechanism for the brain and that this was the route by which some substances entered the brain. This subsequently led to the use of the term “Weg über den Liquor,” although not by Goldmann or by von Monakow to whom it is sometimes attributed.

But Goldmann clearly thought that the choroid plexuses had a protective role in preventing entry of toxic materials into the brain: “Der Plexus vermag andererseits die Zerebrospinalflüssigkeit und damit die Nervensubstanz vor dem Eindringen von Substanzen zu schützen, die sich bei der direkten Einfuhr in den Subarachnoidealraum als ein schweres Gift für die Ganglienzellen erweisen.” (Goldmann, 1913, p. 54): *On the other hand the choroid plexus is capable to protect the liquor and therefore also the nervous substance against the influx of substances which when directly transferred into the subarachnoid space are a strong toxin for the ganglionic cells*. He then summarizes his view of the choroid plexus as a protective regulatory mechanism: “Wir kommen somit zu dem Schluß, dass der Plexus ein wichtiger Schutz-und Regulationsmechanismus für das zentrale Nervensystem darstellt, und dass differenten Substanzen Tür und Tor für ihren Eintritt in das zentrale Nervensystem geöffnet werden, wenn die physiologische Eintrittspforte, der Plexus chorioideus, umgangen wird und solche differente Stoffe durch die Lumbalpunktion in eine direkte Berührung mit den Nervenelementen gebracht warden.” (Goldmann, 1913, p. 55): *Thus we conclude that the plexus is an important protective-and regulatory mechanism for the CNS and that various substances will freely enter the CNS if the physiological gatekeeper, the choroid plexus, is circumvented by a lumbar puncture, those substances will come into direct contact with the nervous elements*. However it should be noted that Goldmann makes no mention of any protective function of cerebral blood vessels.

## Route of entry from blood into brain

Stemming from the studies of Goldmann (1909, 1913) described above and supported by von Monakow (1921) there came the suggestion that the choroid plexuses, rather than the cerebral blood vessels, were the CNS protective mechanism and the primary route of entry of nutritive substances into the brain (described by the term “Weg über den Liquor” by later authors). However, this proposed nutritive function of the choroid plexus seems to have been current earlier than this (see Meek, 1907). Stern in particular initially supported this idea in her interpretation of extensive studies of entry of a wide range of molecules from blood into CSF (Stern and Gautier, 1921) and brain (Stern and Gautier, 1922) in which she emphasized the importance of the choroid plexuses in this role. Davson (1956, 1976) considered this an untenable hypothesis and dismissed the idea as “intellectually inadequate” as it did not take account of substantial vascular circulation of the brain parenchyma. This view was shared by Friedemann (1942): “It is utterly unlikely that in an organ of the vital importance of the brain the capillaries should lack their chief uses, namely, mediation of the exchange between blood and tissue and adaptation of blood supply to functional needs” (Friedemann, 1942, p. 126). The idea was also dismissed by several other German authors (e.g., Friedemann and Elkeles, 1931, 1934; Walter, 1934). However, this dismissal is not justified, as these authors have ignored or miss-cited Stern and Rapoport's short papers (e.g., Stern and Rapoport, 1928a,b) from which it is clear that they believed in the existence of two routes of entry from blood into brain: one directly across the blood vessels (endothelial blood-brain barrier) and the other via the choroid plexuses (Goldmann and Stern's blood-brain barrier, now referred to as the epithelial blood-cerebrospinal fluid-barrier). For example, Stern and Rapoport (1928a) reported that in animals subjected to carbon monoxide poisoning, dyes entered brain tissue directly across the cerebral blood vessels, but only sodium ferrocyanide could be detected in the CSF, which was interpreted as suggesting two different routes of entry into the brain, one (for trypan blue or similar colloids) at the vascular endothelial cells of brain capillaries, the other (for sodium ferrocyanide) via the plexuses.

In Geneva Lina Stern, who was a physiologist and biochemist, did not have anybody in her laboratory to perform a thorough post-mortem histology of the brain of the experimental animals. In Moscow she found a competent neuropathologist/histologist Yakov Rapoport, who could localize which cells in the brain had been colored by the injected trypan blue or had been revealed by the Prussian blue reaction. His contribution was therefore of great importance. Unfortunately their cooperation came to an end in the early 1930's. It seems likely that the two disagreed on the respective roles of the blood-CSF and blood-brain barriers.

In contradistinction to her previous short papers with Rapoport, on the “two barriers model,” Stern in a short paper (1934) focused her attention on the blood-CSF barrier (which she calls the *barrière hémato-encéphalique*). Stern admits that the use of dyes or of other yes-or-no biological markers to assess the permeability of the barrière hémato-encéphalique (blood-CSF barrier) is open to criticism. “En effet il n'est guère possible de tirer des conclusions sur le comportement du liquide en partant simplement de la pénétration ou de la non-pénétration d'une substance donnée présente [dans le plasma sanguin].”: *Actually the composition of the liquid cannot be directly deduced from the passage or non-passage of a substance present in blood plasma*.

According to Stern (1934), the barrier has dual roles of protection and metabolic regulation of the brain. She also introduces the notions of barrier *selectivity* and barrier *resistance*. Thus in this 1934 paper, which was one of the last published in French, Stern makes a very clear statement about the importance of the blood-brain barrier in not only providing protection for the brain but also an appropriate physico-chemical environment for normal brain function: “Partant de l'idée que le liquide céphalo-rachidien represénte le milieu nutritif immédiat des éléments nerveux cérébro-spinaux et que la constance relative de la composition chimique et de l'état physico-chimique de ce milieu est une condition *sine qua non* du fonctionnment normal des centers nerveux, nous avons attributé un rôle de premier plan à l'activité de cet appareil régulateur que représente la barrière hémato-encéphalique.”: *Starting with the idea that the CSF represents the immediate nutritive medium for the cells of the brain and spinal cord and that the relative constancy of the chemical composition and physico-chemical state of this medium is the* sine qua non *for normal function of the CNS we have attributed a primary role for this regulatory activity to the blood-brain barrier*. The concept of the blood-brain barrier providing mechanisms that control the internal environment of the brain is a major physiological concept that was decades ahead of its time and really only began to be properly accepted and investigated with the advent of radioactive tracers in the 2nd half of the 20th Century as outlined below.

Stern (1934) does not mention the relative importance of the cerebral vessels (blood-brain barrier) and choroid plexuses (blood-CSF barrier) in these dual functions of protection and regulation of the internal environment of the brain. This remains a technical difficulty to this day. It does seem highly unsatisfactory that later authors did not take account of Stern's direct studies on permeability of the cerebral blood vessels under both normal and pathological conditions. Another extremely important point made by Stern (1934) was her insistence on the necessity of appreciating that the barrier does not have uniform properties with respect to the wide range of molecules that she studied in earlier works: “… le fonctionnement de la barrière ne présente pas un caractère uniforme vis à vis de toutes les substances examinées…”: *the function of the barrier is not uniform with respect to all substances studied*. This is an extremely important point, which is often still not realized to this day.

In contrast to the adult brain, in the developing brain it is now clear that the choroid plexuses are indeed the main portal of entry into the brain (Johansson et al., 2008). As will be recounted below, after returning from Geneva to Moscow in 1925 (Dreifuss and Sigrist, 2009) Stern made important contributions to the study of brain barriers in developing animals of a number of species in a series of short papers published in the period 1927–1929. If more notice had been taken of Stern's papers, the longstanding belief in the absence of the blood-brain barrier in the fetus and newborn might have been short-lived.

## Early dye studies in immature animals

The important paper of Wislocki (1920), discussed above and illustrated in Figure 1A, was rarely cited in most of the literature of the 20th Century, perhaps because it did not fit with the prevailing belief in immaturity of the blood-brain barrier. Even King (1939), who published with Wislocki, does not mention the seminal 1920 paper preferring instead to cite Behnsen (1927) as evidence of barrier immaturity; the deficiencies of the latter study are discussed next. These are perhaps the most cited in support of immaturity or leakiness of the blood-brain barrier (Behnsen, 1926, 1927) particularly his 1927 paper (e.g., Spatz, 1934; King, 1939; Friedemann, 1942; Davson, 1967). In the 1926 paper Behnsen refers to sealing (“Abdichtung”) of the barrier in the postnatal animals, and writes in the summary on page 1146 that the blood brain barrier is considerably more leaky (original: “erheblich durchlässiger”) than in adult mice as staining with trypan blue appears in young animals more intense generally and also in different places of the CNS compared to the staining pattern of adults. However, in some slightly handicapped adult mice Behnsen also found additional areas in the brain which were stained, that he called those weak spots (orig. “schwache Stellen”), besides those where apparently no barrier exists (orig. “offene Stellen”) and puts this in contrast to Goldmann's view that the central nervous is entirely sealed from dye (Goldmann, 1909, 1913). He also suggested: *The fact that the blood-brain barrier in the young is more leaky may also relate to immaturity of tissues and cells and a different level of cellular maturity*. (orig.: “Im Speicherverhalten der nervösen Zellen zeigen sich Unterschiede, die … auf einen verschiedenen Reifezustand der Zellen zu beziehen sind,” Behnsen, 1926. p. 1146). It may be that Behnsen's finding of dye in some parts of the brain was due to the use of an excessive amount of dye (three injections of 1% trypan blue).

Behnsen's paper of 1927 is longer and more detailed and the results and interpretations differ in some respects to the 1926 paper. He speaks more cautiously about an increasing sealing of the barrier during postnatal development and points out that even in adult mice staining can be found in the brain due to local penetration of dye. He commented: “*The sites of dye penetration in the adult animals correspond to the maximal storage in young mice. They are located 1. In the region of Retzius'ependymal wedge in the medulla oblongata; 2. At the attachment sites of the choroid plexus; 3. In the infundibular recess*” (Translation of orig.: “Die Farbstoffdurchtrittstellen der erwachsenen Tiere entsprechen den Speicherungsmaxima der jungen Mäuse…”).

Behnsen (1927) himself does not appear to state unequivocally that the barrier is immature in the newborn and his experiments have a number of curious features. As was the case for many barrier studies in the early part of the 20th Century, they involved multiple injections of trypan blue into postnatal (2–3 week old) and adult mice. He reported that areas of the brain that stain for trypan blue at postnatal ages were more extensive than in the adult, but they are located at the same sites, which Behnsen calls “Grenzflächen” i.e., interfaces/membranes between brain and bloodstream or even more generally used by Behnsen: membranes involved in barrier functions are of the same structure in young and adult animals, they only differ gradually in their function. “Die Übereinstimmung der Speicherstellen der erwachsenen Tiere mit denen der jungen macht es sehr wahrscheinlich, daß die Grenzflächen im erwachsenen Zustand, nur graduell verschieden, dieselben örtlichen Bauunterschiede aufweisen, wie sie das Speicherbild für junge Tiere offenbarte.” (Behnsen, 1927, p. 570): *The identical storage sites (of dye) in adult animals and in young ones makes it highly likely that the interfaces (“Grenzflächen”) in the adult condition differ only gradually, but are otherwise of the same structure as revealed by the dye storage of young animals*.

**Figure 3:**
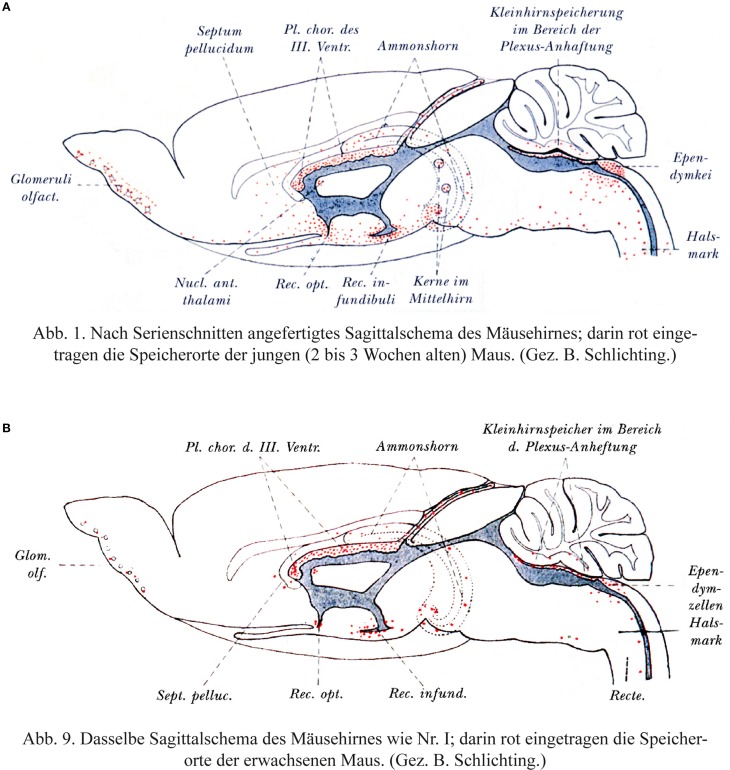
**Behnsen's much cited trypan blue experiments. (A)** Brain from postnatal mouse injected systemically 3 times with trypan blue at P4–P14. **(B)** Adult mouse brain following systemic injection of trypan blue. Note that as described by Behnsen the dye staining in the postnatal brain is in the same regions as in the adult brain, but more extensive. This is probably a visual artifact due to the use of an outline of an adult mouse brain for both ages. In postnatal mouse brain (P14) the cerebral cortex and cerebellum are less developed than illustrated here. Note also that in the postnatal brain there does not appear to be any dye staining of the neocortex-the region of the developing brain with the least mature blood vessels. From Behnsen (1927).

This is illustrated in two figures reproduced here in Figure 3. The distribution of trypan blue is illustrated in sagittal sections of mouse brain from the two ages. However, it is the same line drawing of an adult brain that is used for both ages. This gives the misleading impression that the distribution of the dye in the postnatal brain was wider than it probably was in the actual microscopical slides. This is because the neonatal brain is less well developed particularly with respect to the neocortex. **It is also notable that there is no staining indicated in the postnatal neocortex as this is the region with the least well developed vasculature in the postnatal period compared to the rest of the brain; were the blood-brain barrier to dye to be immature, this is precisely where dye staining would be expected to be apparent**. Bakay (1956) commented that although Behnsen's conclusions are frequently misquoted “they do not reveal a generalized increase in permeability of the barrier.”

Behnsen's experiments were repeated in careful detail by Moos and Møllgård (1993). They studied the distribution of trypan blue and Evans blue, a dye that superseded trypan blue from about mid-20th century for blood-brain barrier studies. Moos and Møllgård (1993) used a range of concentrations of the dyes some of which exceeded the binding capacity of plasma albumin to which these dyes bind. Binding of dyes to plasma albumin appears to have been overlooked by the blood-brain barrier field until the observations of Tschirgi (1950), although Ehrlich himself commented that the lack of some of his small molecular weight dyes in urine, was probably accounted for by binding to plasma albumin (Ehrlich, 1885). Also, Stern and colleagues (e.g., Stern et al., 1929) made a distinction in guinea pig embryos and neonatal animals of various species between the blood-brain barrier permeability of low molecular weight sodium ferrocyanide and the lack of penetration of trypan blue, which they described as a colloid. Whether this description reflects knowledge of protein binding or aggregation of the dye in blood is not clear. However, the difference between entry of small lipid insoluble molecules (sodium ferrocyanide in early qualitative experiments and sucrose and inulin in later quantitative experiments) and the lack of entry of trypan blue turned out to be a fundamental distinction in properties of blood-brain and blood-CSF barriers, as will be described below.

Stern et al. (1924) studied the entry of sodium ferrocyanide from blood into CSF in various species at different stages of development. They reported that in the guinea pig the barrier to this molecule was already as in the adult, but in species born at a less mature stage of development (rats, mice, cats) sodium ferrocyanide could be detected in the CSF. Stern and Peyrot (1927) extended the earlier study of Stern et al. (1924) examining entry sodium ferrocyanide into the CSF. In guinea pig fetuses this marker did enter the CSF, but as reported earlier not in the newborn of this species. They confirmed the entry of sodium ferrocyanide in the newborns of rats, mice and cats and in addition rabbits.

Stern and Rapoport (1928a) studied the injection of dyes (trypan blue and Congo red) in neonates of various species. They distinguished clearly between a barrier at the level of the vascular endothelium and the choroid plexus epithelium in dye studies where cellular staining following dye injections, but unlike other authors they thought this was a post mortem phenomenon. In a following year using the same dyes Stern et al. (1929) made the important point that it was necessary to limit the amount dye injected. They stressed that in experiments in adults and developing animals using similar amounts of dye, no dye entered the CSF. But if the amounts were increased substantially (30–40 times than used normally) then staining of the CSF occurred.

Another dye study that is sometimes cited as evidence of barrier immaturity in neonates (e.g., Ribatti et al., 2006) is that of Penta (1932). Penta injected multiple doses of trypan blue or trypan red into postnatal rabbits and cats. Penta seems to have considered that the results of Behnsen and Stern were similar, although as described above Stern et al. (1929) found no entry of trypan blue in the brains of developing animals providing not too much dye was injected. Penta summarized his results as showing that the nervous system of neonates is colored more intensely than that of adults and staining occurred in the regions where the mesenchyme penetrates into the neuraxis. As in many of the adult studies using trypan blue and against the advice of Stern (Stern et al., 1929) Penta mainly used multiple large doses of trypan blue and many of the animals died from the toxic effects of the dye; therefore staining of the brain was hardly surprising. However, Penta did describe one experiment in which much less dye was used in which no staining of the brain (apart from the choroid plexus) occurred, thus confirming Stern's results, without Penta apparently realizing it.

There seems little doubt that at least part of the problem with many early dye studies, both in developing animals and adults, was that the large amounts and multiple injections used led to toxic effects. This might be indicative of a greater fragility of brain barriers in immature animals but should not be interpreted as evidence of an immature or absent blood brain barrier.

The binding of dyes to albumin in plasma was investigated systematically by Moos and Møllgård (1993). They showed that dye only entered the brains of neonatal (one day old) mice and rats if the albumin binding capacity was exceeded, except in regions of the brain known to be devoid of a blood-brain barrier. For concentrations of dye (trypan blue, 1–2%; Evans blue, 0.5–1.0%) that were shown not to exceed the binding capacity of albumin in plasma, neither dye penetrated into the brain by exudation across cerebral vessel walls in either P10 or adult mice, although there was some staining of choroid plexus epithelial and cerebral endothelial cells. At higher concentrations of the dyes (trypan blue, 2–4%; Evans blue, 2%) at which there was a substantial level of free dye, 60% of neonates and 20% of adults died, with higher proportions at the higher concentrations of dye. Those animals that survived showed various signs of neurotoxicity. Toxic effects of trypan blue had been reported earlier (e.g., Behnsen, 1927; Penta, 1932) but the significance of this in interpretation of barrier studies with dyes seems to have been overlooked by all but Stern who urged caution in the amount of dye to be injected into neonatal animals (Stern et al., 1929).

## Later studies of dyes, albumin and other markers in immature animals

**Figure 4:**
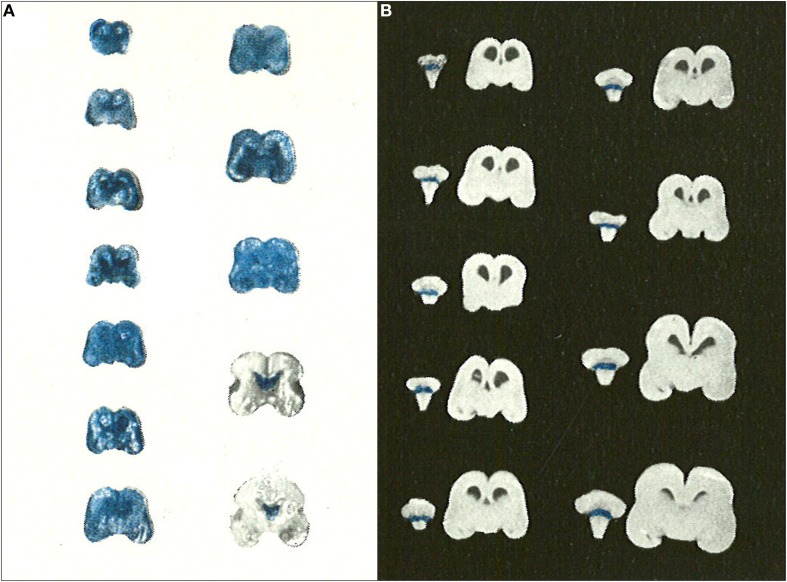
**Gröntoft's dye experiments with postmortem aborted human fetuses. (A)** Human fetuses (14 cm-18 weeks to 30 cm-31 weeks gestation) injected with trypan blue solution 30 min after caesarian section delivery. **(B)** Human fetuses (14 cm-18 weeks to 30 cm-31 weeks gestation) injected with trypan blue solution 10 min after caesarian section delivery. Note that for the shorter period of anoxia none of the brains stained with trypan blue indicating intact blood-brain barrier to trypan blue even in immature human fetuses. Reproduced with permission from Gröntoft (1954), © Wiley.

The use of dyes to study blood-brain barrier permeability in immature animals began to die out in the second half of the 20th Century. This is in contrast to the adult blood-brain barrier field, which continues to place a heavy reliance on dyes, particularly Evans blue, for assessing blood-brain barrier integrity. All of the studies published after 1950 agreed in showing that dyes (usually trypan blue) did not enter the developing brain no matter what age was investigated. The most important of these studies is probably that of Gröntoft (1954) who did experiments in both human fetuses (recent abortions) and newborns. Gröntoft emphasized that the condition of the fetuses was very important in determining whether or not the dye entered the brain. In a series of carefully conducted experiments he showed clearly that when trypan blue was injected as soon as possible after separation of the placenta in human fetuses of a wide range of gestational age, the dye did not stain the brain as a whole, but only those areas (choroid plexus and circumventricular organs) that are outside the blood-brain barrier (Figure 4); this was the case even in a very immature embryo of 5 cm in length (approximately 10 weeks post conception). However, when the dye was injected at progressively longer intervals after placental separation of the fetuses (10–30 min depending upon the gestational age) then the whole of the brain stained. Gröntoft carried out similar well-controlled experiments in rabbit fetuses with the same findings.

Grazer and Clemente (1957) injected by various routes solutions of trypan blue into rat embryos from E10.5 to birth. They found no evidence of dye penetration into the brain. Millen and Hess (1958) injected i.p. two doses, 24 h apart, of either trypan blue or sodium ferrocyanide, into neonatal (P0–P8) and adult rats and the animals were examined after 30 min. No staining of the brain for either marker was identified in the neonatal animals apart from the restricted regions (outside the blood-brain barrier) that also stained in the adult. Similar results were reported by Olsson et al. (1968) who injected fluorescently labeled bovine albumin intravascularly in rat embryos as early as E15, compared with neonates and adults.

In the second half of the 20th Century there was a switch from using dyes to using horseradish peroxidase (HRP) as a test of barrier integrity in the developing brain. HRP has a number of problems that are not always well recognized by those that use it. In the context of development the main problem is that, as with the dyes in earlier experiments, too large a volume or concentration of HRP was injected, thus damaging fragile cerebral blood vessels and giving the appearance of a “leaky” blood-brain barrier (see Table 1). Several other authors reported the entry of HRP into brains of very immature embryos, for example chick embryos (Delorme et al., 1970; Delorme, 1972; Bertossi et al., 1992). However, insufficient detail of exactly what was injected makes it difficult to evaluate the results of these studies. The finding that HRP entered the brain at different embryonic ages in the same species or at different stages of brain development in different species suggests that the findings from these studies are likely to have been artefactual. There have been a few reports of transendothelial passage of markers such as HRP in developing brain (e.g., in tubulovesicular structures, Lossinsky et al., 1986) which may explain the entry of the marker into immature brain in some experiments, as lack of pinocytotic vesicles is a characteristic of adult cerebral endothelial cells.

**Table 1:** **Injection experiments used as test of blood-brain barrier integrity in fetal and neonatal animals**.

**Authors**	**Species**	**Age◊ (days)**	**Body weight (g)^a^**	**Injection vol^b^ (% total blood vol)**	**Increase in plasma protein concentration^c^**	**“Leaky” barrier claimed•**	**Injected material^d^**
Grazer and Clemente, 1957	Rat	E10.5	<0.06	Not stated	–	No	Trypan blue i.p./i.v.^b^
Olsson et al., 1968	Rat	E15	0.14	<5%	–	No	Fluorescein- albumin, Umb.A.
Delorme et al., 1970	Chick	E4.5	0.1	Not stated	3%	Reaction product in neuropil ECS until E10	HRP i.v.
Wakai and Hirokawa, 1978, 1981	Chick	E8	0.5	10%	500%	Yes	HRP i.v.
Dziegielewska et al., 1979, 1980	Sheep	E60	60	7.5%	20%	No	Human plasma protein i.v.
Lossinsky et al., 1986	Mouse	Newborn	1.4	35%	2%	Yes, “massive leakage”	HRP i.v.
Roncali et al., 1986	Chick	E6	0.34	>150%	50%	“Unimpeded extravasation”	HRP intracardiac^e^
Risau et al., 1986	Mouse	E13	0.08	250%	1% increase	“Fully permeable”	HRP intracardiac^e^
Vorbrodt et al., 1986	Mouse	Newborn	1.4	100%	200–300%	Yes^**^	HRP i.v.
Stewart and Hayakawa, 1987	Mouse	E15	0.26	Not stated	100%^*^	Yes	HRP i.p.^f^
Dziegielewska et al., 1988	Tammar wallaby	Newborn	0.50	10–20%	25–50%	No	HRP/HSA i.v.
Hulsebosch and Fabian, 1989	Rat	Newborn	5.0	50%^*^	>400%^*^	IgG penetration into neuraxis	IgG i.p.^b^
Dermietzel et al., 1992	Rat	E14.5	0.14	100–150%	>100%	Yes	HRP or 40 kDa dextran intracardiac^d^
Ribatti et al., 1993	Chick	E6	0.36^a^	>150%	50%	“Massive diffusion”	HRP or Evans blue, intracardiac
Xu and Ling, 1994	Rat	Newborn	5.0	10%	65%	Yes	Ferritin solution i.v.
Knott et al., 1997	Opossum	P3	0.20	8%^*^	0.5%^*^	No	Human albumin i.p.

a *Body weights and circulating blood and plasma volumes for chick embryos from Romanoff (1967) and Davis and Garrison (1968). Body weights of fetal rats from unpublished data. Body weights of opossum from Saunders et al. (1989) and unpublished. Weight of youngest animal given*.

b *The authors' injection volume when stated has been compared to estimates of circulating blood volume (10% of body weight and allowing a factor of 2 for placental blood volume in the case of fetuses and for extra-embryonic blood volume in chick embryos, although it is doubtful if under the conditions of some of these experiments, much mixing with fetal placental blood would have occurred*.

c *The actual circulating blood volume in sheep fetuses has been measured (Dziegielewska et al., 1991). The effect of the amount of injected protein on the plasma protein concentration is also estimated when sufficient information is available. Plasma protein concentrations from Birge et al. (1974, chick embryo) and Dziegielewska et al. (1981, rat fetus)*.

d *Information is not usually available about the speed of injection nor about the likely distribution volume (e.g., in experiments involving intracardiac injection*.

e *In which the normal circulation may not have continued or i.p. injection*.

f *In which it is not clear what proportion of the volume and protein will have entered the circulation)*.

*^◊^Youngest age studied. E is embryonic age from day of conception*.

* *Depending on proportion entering circulation*.

** But “not detected at E19.”

*•Not all authors of experiments in which marker entered brain tissue from blood vessels describe this as a “leak” or due to immaturity*.

## Entry of small molecular compounds into the brain and CSF of immature animals

Lina Stern and her colleagues published a preliminary report of studies in a range of species of developing animals (Stern et al., 1924). This was followed by a series of brief papers in the period 1927–1929 on the permeability of the “barrière hémato-encéphalique” to sodium ferrocyanide and several other low molecular weight compounds in fetal guinea pigs and newborns of several species (Stern and Peyrot, 1927; Stern and Rapoport, 1928a,b; Stern et al., 1929). Following injection into fetuses via the uterine horn of pregnant guinea pigs early in gestation, sodium ferrocyanide could be demonstrated by the Prussian blue reaction in the CSF and CNS tissue. However, when sodium ferrocyanide was injected into term embryo or newborn guinea pigs its presence could not be detected in either CSF or CNS tissue. In contrast, when sodium ferrocyanide was injected into the newborns of rabbits, rats, mice, dogs, and cats it could be demonstrated in CSF and nervous system tissue and for several days into the neonatal period. Stern and Peyrot (1927) correlated the cessation of sodium ferrocyanide entry into CSF and nervous tissue with the time of eye opening and suggested that this was the time when the blood-brain barrier (“barrière hémato-encéphalique”) to certain substances reached a “normal value,” presumably meaning that it reached the adult condition. They concluded that in species born at less mature stages of development (which they suggested probably included human newborns) the “activity” of the barrier was insufficient to protect the CNS against many substances circulating or introduced into the blood.

These qualitative observational results with a small molecule (sodium ferrocyanide 304 Da) foreshadowed quantitative experiments with radiolabelled small molecules such as mannitol (182 Da), sucrose (342 Da), and inulin (5200 Da) in newborns of rats (Ferguson and Woodbury, 1969; Woodbury et al., 1974; Johanson, 1989; Habgood et al., 1993) and fetal sheep (Evans et al., 1974; Dziegielewska et al., 1979), which showed higher CSF/plasma and brain/plasma ratios for the these molecules in immature animals. Although initially interpreted as indicating greater permeability of the blood-brain and blood CSF barriers, it became apparent from measurements of CSF secretion in embryos (Evans et al., 1974; Fossan et al., 1985) and newborns (Bass and Lundborg, 1973; Johanson and Woodbury, 1974) and increase in volume of CSF in rat embryos (Johansson et al., 2006, 2008) that the explanation is more likely to be that the much lower turnover (sink effect) of CSF in the developing brain allowed a greater accumulation of marker in CSF and brain (Bass and Lundborg, 1973; Johansson et al., 2008). However, it took the development of the use of small molecular weight markers that could be both quantified and visualized at the electron microscopic, EM level to show that the route of penetration from blood to CSF of these small markers was via an *intracellular* route in a subpopulation of epithelial cells in the choroid plexuses (Ek et al., 2001, 2003, 2006). These cells appear to be responsible for the transfer of inert dextrans and several plasma proteins from blood into CSF both in the developing and adult brain (Dziegielewska et al., 1991; Liddelow et al., 2009, 2011a). The absolute number of cells involved in this transfer actually increases with age as the choroid plexuses develop, but the CSF/plasma ratios for dextrans and proteins fall, because of the increased turnover of CSF and a larger space into which these markers are diluted (Johansson et al., 2006, 2008).

## Kernicterus in the newborn

One of the persistent beliefs about the blood-brain barrier in the immature organism is that the toxic effects of unconjugated bilirubin that accumulate in the blood of newborn infants suffering from erythroblastosis, generally due to Rhesus blood group incompatibility, are due to “immaturity” of the blood-brain barrier in the newborn. This is a suggestion generally attributed to Spatz (1934) e.g., Broman (1941); Davson (1967), and Lee (1971). Others stated that this was the case but without attribution (e.g., Ganong, 1993; Cotran et al., 1994). Bakay (1956) expressed the view that “the phenomenon of kernicterus appeared to offer irrefutable proof of this theory [that the blood-brain barrier in the newborn is immature].” In his later review Bakay is more circumspect and suggests that the causation of kernicterus has many components of which an immature blood-brain barrier might be one (Bakay, 1968).

**Figure 5:**
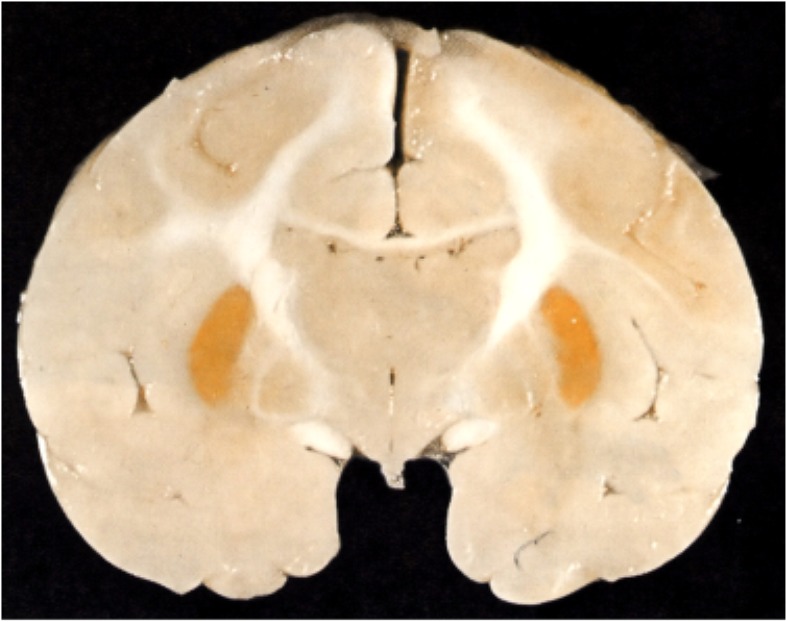
**Kernicterus in brain of neonatal monkey with jaundice**. Note staining of putamen (one of the basal ganglia), which does not occur in adult brain as jaundice in adults, is generally due to high levels of conjugated bilirubin to which the blood brain barrier is impermeable. From Windle (1969).

Most of Spatz's lengthy paper is taken up with describing and repeating some of Behnsen's (1927) experiments with trypan blue. But he does have a short section on icterus (jaundice) in the adult, with an illustration showing that yellow pigment of bilirubin was confined to dura. He compared this (without illustration) to brain damage (kernicterus) that only occurs in the newborn with the deposition of bilirubin in the brain, characteristically in the basal ganglia and brain stem (Figure 5). He does not seem to distinguish between conjugated bilirubin, which accumulates in the circulation of adults sometimes to very high levels, and unconjugated bilirubin that accumulates in newborns because the activity of the conjugating liver enzyme, glucuronyl transferase, is very low at birth and normally increases during the first days of life. These two forms of bilirubin had been identified earlier (van den Bergh and Müller, 1916). What was not known in the 1930s was that unconjugated bilirubin binds tightly to plasma albumin and a key factor (but not the only one) in the occurrence of kernicterus is when the binding capacity of albumin for unconjugated bilirubin is exceeded; this form of bilirubin which is highly lipid soluble (in contrast to conjugated bilirubin) enters the brain without restraint.

It is noteworthy that clinicians, who have had to deal with the problem of kernicterus in the newborn for many years, rarely seem to have invoked the state of maturity of the blood-brain barrier in the neonate as a major factor in the occurrence of kernicterus (e.g., Iskander et al., 2014), although it has occasionally been mentioned (e.g., Zuelzer and Mudgett, 1950; Spiegel-Adolf et al., 1954; Ernster et al., 1957); rather they developed clinical treatments aimed at reducing the level of unconjugated bilirubin in infants who were at risk: exchange transfusion (Allen et al., 1950; Gerrard, 1952) barbiturate induction of glucuronyl transferase and phototherapy (Lucey, 1971) as well as prevention by immunization of Rh-ve women against the D blood group antigen (Clarke, 1989). But the linking of blood-brain barrier immaturity to kernicterus has persisted amongst other groups of scientists and clinicians for decades after Spatz (1934). A good example is a physiologist, (Ganong, 1993) who stated that: “Bile pigments do not enter the brain in the adult but in the fetus and newborn infant the blood-brain barrier is not developed.” Following correspondence with one author (NRS) in later editions Ganong (1999) modified this to state that “the bilirubin penetrates the brain of infants with erythroblastosis at least in part because the blood-brain barrier is more permeable in infancy”; he was not willing to take account of the effect of binding of unconjugated bilirubin to plasma albumin. Similar wording has been retained by the new authors of Ganong's Review of Medical Physiology (Barrett et al., 2012) following his unfortunate demise. Some aspects of bilirubin neural toxicity are discussed by Palmela et al. (submitted).

Some pathologists have also considered that kernicterus is due to immaturity of the blood-brain barrier (Cotran et al., 1994). A detailed consideration of kernicterus is beyond the scope of this review. But comprehensive papers are available both on the pathogenesis and treatment particularly by Wennberg and his colleagues (e.g., Wennberg, 2000; Ahlfors and Wennberg, 2004; Wennberg et al., 2006) and in Volpe (2008). However, it is becoming increasingly clear that the mechanisms involved in whether or not kernicterus occurs in newborns are much more complex than the absolute level of unconjugated bilirubin and the binding capacity of albumin in plasma. These complexities have been reviewed by Ghersi-Egea et al. (2009) and include the finding that bilirubin is a substrate for several ABC efflux transporters, which would thus be expected to contribute to limiting the entry of bilirubin into the brain.

## Experiments with radioisotopically labeled molecules in developing animals

The introduction of radiolabelled markers was a major advance in experimental approaches to understanding blood-brain barrier mechanisms. The use of inert markers of permeability such as sucrose and inulin has been mentioned above. In this section we shall deal briefly with experiments involving the use of P^32^ and amino acids labeled with ^3^H or ^14^C. Compared to inert markers there are potential problems with metabolically active radiolabelled molecules such as amino acids. Thus it needs to be shown that the radioactive tag is still attached to the molecule and that the measured radioactivity is due to transfer into the brain separate from its metabolic incorporation, for example into proteins.

### ^32^Phosphorus

Bakay (1953, 1956) studied the uptake of ^32^P into brain and other organs following parenteral injection into pregnant (3rd–4th week), newborn, up to 7 weeks postnatal rabbits and in the adult. Animals were killed 24 h after injection and various organs dissected out. In some cases radioautographs of the heads or brains of fetuses were prepared. The results showed a much higher uptake of ^32^P in the younger the animals. In fetuses and newborns the brain/blood ratio was >1, compared to about 0.3 in the adult, which Bakay (1953) interpreted as indicating that the vascular permeability in the fetal brain is increased. He discusses whether the increased permeability reflects a leaky, incompletely formed blood-brain barrier or increased metabolism and faster turnover in the developing nervous system. He concluded that neither explanation was satisfactory. Bakay (1953) observed that in the radioautographs of fetal brain the distribution of ^32^P was almost uniform whereas in the brain of the mother it was concentrated in the choroid plexuses and the lining of the ventricles. Rather puzzlingly he took this as evidence that there is a blood-brain barrier to ^32^P in the fetus but that its permeability is greater than in the adult.

### Amino acid transport

In early experiments with radiolabelled amino acids there was a similar finding of a much greater entry of some (but not all) into the developing than adult brain of several species (Himwich et al., 1957; Roberts et al., 1959; Purpura and Carmichael, 1960; Lajtha and Toth, 1961; Seta et al., 1972; Baños et al., 1978) but as with ^32^P it was not possible to distinguish between cerebral endothelial cell transport and metabolic incorporation into brain tissue in accounting for the greater uptake in developing brain. Some authors interpreted such results as indicating “lesser effectiveness” of the blood-brain barrier for amino acids in young animals (e.g., Kuttner et al., 1961; Lee, 1971). The problem of determining cerebral transport of amino acids across the blood-brain barrier without the complication of metabolic contamination of brain samples was solved by Oldendorf (1971) who developed a short pass technique in which exposure of amino acid or other metabolically active molecules to the brain from the cerebrovascular circulating blood was limited to one circulation time, thus being too quick for significant metabolic incorporation to occur. The technique was adapted for newborn and postnatal animals by Oldendorf's students (Braun et al., 1980; Cornford et al., 1982; Pardridge and Mietus, 1982) and by Lefauconnier and Trouvé (1983), Al-Sarraf (2002), and Al-Sarraf et al. (1997). These experiments showed that many amino acids and other metabolically active compounds were transported into developing brain at much higher rates than in the adult; this was interpreted as reflecting the greater metabolic demand of the developing brain rather than immaturity of the blood-brain barrier. However, some authors have continued to suggest that greater uptake may reflect barrier immaturity (e.g., Watson et al., 2006).

Thus it has been clear for sometime that transport mechanisms at the brain barrier interfaces determine the composition of the internal environment of developing brain and supply essential nutrients and other molecules important for growth and differentiation of the brain. What has been lacking until recently is information on the presence and expression of specific transporters in the brain barrier interfaces. This is now available from expression studies of isolated cerebral endothelial cells from neonatal mice (Daneman et al., 2010a) and from choroid plexuses of fetal mice and rats compared to the adult (Liddelow et al., 2012, submitted). Many of these so called Slc transporters are expressed at higher levels in the developing brain compared to the adult, thus accounting for the earlier observations of higher entry of specific amino acids into the developing brain. There are differences in the expression patterns of these Slc transporters in the cerebral endothelial cells and in the choroid plexus epithelial cells and many more are expressed at higher levels in the fetal choroid plexus (see reviews by Saunders et al., 2013 and Liddelow et al., submitted). This reflects the greater importance of the choroid plexuses for transport into the brain compared to the vasculature at very early stages of brain development. The plexuses develop much earlier than the blood supply (Johansson et al., 2008). The findings also help to explain a number of important observations on developmentally different effects of amino acids on brain function. For example it was reported many years ago that glutamate is toxic to the brain if administered in the neonatal period (Olney and Ho, 1970) which some attributed to “immaturity” of the blood-brain barrier (Viña et al., 1997). However, it can now be seen that the barrier contribution to toxicity is much more likely to be due to greater transport of glutamate by e.g., Slc1a4 (see Saunders et al., 2012).

## The paracellular pathway and tight junctions in barrier interfaces in the developing brain

The concept of the paracellular pathway is an important tenet of epithelial biology that was extended to cerebral endothelial interfaces. It arose from some ingenious experiments of Frömter and Diamond (1972). These authors measured transepithelial resistance in a variety of epithelia *in vitro* by passing a microelectrode over the external surface of the epithelium. They observed that when the electrode tip was over the region of the border between adjacent cells there was a marked drop in resistance across the epithelium. Frömter and Diamond proposed that this indicated a low resistance pathway across the epithelium for water and ion flow. They subsequently extended this concept to include the paracellular pathway as the route by which small lipid insoluble molecules such as sucrose crossed epithelia (Diamond, 1974). The physical basis for the paracellular pathway is the junctional complex between adjacent cells and in particular the tight junction component of the complex (Brightman and Reese, 1969). However, there are some important limitations to this interpretation of the sites of low resistance across epithelia. Firstly the dimensions of the tip of the microelectrodes used by Frömter and Diamond was about 5 μM compared to the intercellular space at the level of tight junctions, which is measured in nanometers, if not zero where adjacent cell membranes are fused. Thus an alternative explanation for the low resistance pathway is that there is an intracellular pathway close to the border of the cell membrane between adjacent cells.

Diamond (1974) seems to have acknowledged this possibility as he suggested that in some epithelia the low resistance pathway might be due to “leaky” cells, although he thought this would be exceptional. Secondly, water and ions cannot yet be visualized with sufficient resolution for their route across epithelia to be defined. Until recently this was also the case for low molecular weight compounds such as sucrose and inulin; however, this problem has been solved by the use of biotin labeled small molecular sized compounds that can be visualized at both the light and electron microscopical level. At least in the case of choroid plexus epithelial and cerebral endothelial cells in the South American opossum, *Monodelphis domestica*, the intercellular junctions are impermeable to a compound biotin ethylenediamine that is smaller than sucrose (Ek et al., 2001, 2003, 2006). In addition it was found that biotin labeled markers transfer across a subpopulation of plexus epithelial cells and not between them (Ek et al., 2001, 2003, 2006; Liddelow et al., 2009). These studies require independent replication, preferably in several different species, but they indicate that the earliest vessels growing into the brain are structurally well enough developed for their tight junctions to be impermeable to even very small molecules. These results also suggest that the presence of a low resistance pathway across epithelial cells, rather than the paracellular route, as envisaged by Diamond (1974) as an exceptional situation may indeed exist.

Over many years the results of ultrastructural studies of tight junctions in the cerebral vasculature and choroid plexuses of the developing brain have been conflicting. Two of the earliest ultrastructural studies of the developing mammalian brain barriers were those of Donahue and Pappas, (1961, rat cerebral capillaries) and Tennyson and Pappas (1964, rabbit choroid plexus). Most of their descriptions dealt with subcellular structures, but they do comment on and illustrate what in those days were called terminal bars that were present in the earliest embryos examined (E14). These appear to correspond to structures associated with what were subsequently described as tight junctions (Brightman and Reese, 1969). Particularly considering that the 1960s were early days for electron microscopy of developing brain tissue (which in those days was regarded as very difficult to fix adequately) the micrographs of Donahue and Pappas and Tennyson and Pappas are of high quality and show clearly well formed intercellular tight junctions. Tennyson (1975) published a more comprehensive paper on choroid plexus development in human and rabbit fetuses. It includes illustrations of what she now refers to as “tight junctions” in the earliest rabbit fetuses studied (E15).

Subsequent to the early paper of Tennyson and Pappas (1964) there have been numerous transmission electron microscope studies of tight junctions in the developing brain, mainly in chick embryos (Delorme et al., 1970; Delorme, 1972; Roy et al., 1974) rodents (Stewart and Hayakawa, 1994) fetal sheep (Møllgård and Saunders, 1975, 1977; Møllgård et al., 1976, 1979) but also some in human fetuses (Møllgård and Saunders, 1975, 1986; Virgintino et al., 2004). Most, but not all, seem to agree that the typical appearance of closely apposed cell membranes of adjacent cerebral endothelial cells appears very early in brain development, although some authors (e.g., Stewart and Wiley, 1981) nonetheless conclude that the blood-brain barrier at these early stages is more permeable than in the adult. This was based on citing papers showing greater entry of both metabolically active and inert molecules into the developing brain and a high concentration of protein in CSF (cf. Sections 6, 8, and 11).

Some authors also used HRP in an attempt to delineate the age at which there was a change from blood-brain barrier permeability to one of impermeability. However, insofar as the methodological information is available it seems likely that in many experiments excessive volumes or concentrations of HRP solution or other markers were injected, thus mechanically disrupting the fragile blood vessels (e.g., Wakai and Hirokawa, 1978, 1981; Risau et al., 1986; Stewart and Hayakawa, 1987; Bertossi et al., 1988; Ribatti et al., 1993; and see Table 1). Also HRP has toxic effects and may give rise to anomalous permeability results, even in adult animals (e.g., Mazariegos et al., 1984). Nevertheless Tauc et al. (1984) were able to show that tight junctions in the choroid plexuses of rat embryos at least as early as E14 were impermeable to HRP. These observations were supported by freeze fracture studies showing a similarly early development of tight junction structure.

Møllgård et al. (1976) carried out a quantitative study of freeze fracture tight junction complexity in fetal sheep embryonic choroid plexus. This showed that there were only minor changes in junctional complexity over a large period of development in contrast to reports in the same species of large developmental changes in apparent permeability from blood to CSF for small molecular markers (Evans et al., 1974; Dziegielewska et al., 1979). It should be noted that the large change in apparent permeability was probably mainly due to an increase in CSF secretion rate (see above), but this does not diminish the significance of the finding that the freeze fracture appearance of choroid plexus tight junctions appeared very early in development.

Dermietzel et al. (1977) published a detailed freeze fracture study of the development of intercellular junctions of the chick embryo choroid plexus from E5, the day on which it begins to differentiate. A junctional network of strands was apparent by E6 and by E9 intercellular zonula occludens were more or less complete.

Attempts to correlate expression of individual tight junction genes with some supposed age when cerebral blood vessels stop being leaky, seem doomed to failure unless experiments are conducted under adequate physiological conditions. The findings that different tight junction genes are expressed at different ages and that the junctions become molecularly more complex with age seem more likely to reflect changes in the mechanical properties of tight junctions in the face of increasing hydrostatic pressure (systemic blood pressure) during embryonic and fetal development (Evans et al., 1974; Dziegielewska et al., 1979). The question of whether or not there is a stage of early vascularization of the brain when the blood vessels are leaky is a functional one that can only be answered by permeability experiments conducted under reasonably physiological conditions. In spite of several attempts (e.g., Risau et al., 1986; Mizee et al., 2013; Ben-Zvi et al., 2014, see also Bauer et al., 2014) there is no convincingly demonstrated link between the expression of one particular gene and the supposed sealing of blood vessels entering the CNS tissue, apart from Nitta et al. (2003) who showed increased permeability to small molecules (<800 Da) in claudin 5-deficient mice; this was attributed to tight junction permeability although not confirmed at the ultrastructural level.

## Ionic gradients between CSF and plasma in the developing brain

Some of the most convincing evidence that some critical brain barrier properties develop very early in the embryo comes from studies of ion gradients between CSF and plasma. In the adult the ionic composition of CSF is characteristically different from that of plasma and is also appreciably more stable. This reflects the importance of a homeostatically constant environment that is a prerequisite for normal brain function (Davson, 1967; Davson and Segal, 1996). The gradients are set up by ion pumps in the epithelial cells of the choroid plexuses (Damkier et al., 2013). Similar pumps in the cerebral vascular endothelial cells may also contribute via the extracellular fluid of the brain (Davson and Segal, 1996). An essential structural component of this homeostatic mechanism is the presence of tight junctions between the adjacent cells of the choroid plexus epithelium and cerebrovascular endothelium. Thus in development, if even a single ion gradient is present, it indicates the presence of both a cellular ion pump and functional integrity of the tight junctions, as without the latter a gradient simply cannot be established. In the 1970s–80s there were several studies in a variety of species showing the presence of ion gradients between CSF and plasma (Flexner, 1938; Bito and Myers, 1970; Bradbury et al., 1972; Amtorp and Sørensen, 1974; Sedlácek, 1975; Mitchell et al., 1982; Nattie et al., 1984). More recently gene expression studies have shown that key ion channel and transporter genes are expressed very early embryonic life in the choroid plexuses (Liddelow et al., 2012, 2013).

## Concentration and origin of proteins in CSF in developing brain

One of the pieces of evidence sometimes put forward in favor of immaturity or leakiness of the blood-brain barrier in the developing brain is that the CSF in fetal and newborn animals contains a much higher concentration of protein than in the adult (Adinolfi et al., 1976; Adinolfi and Haddad, 1977; Ramey and Birge, 1979; Adinolfi, 1985; Fishman, 1992). Strictly speaking the composition of CSF is much more determined by movements of molecules across the choroid plexuses rather than entry via the blood vessels and brain interstitial fluid, although clinicians frequently (but erroneously) interpret CSF changes as reflecting changes in permeability of the blood-brain barrier. Since the middle of the last century it has been known that the concentration of protein in human newborn CSF (e.g., Spiegel-Adolf et al., 1954; Nasralla et al., 1958) is higher than in the adult (Davson and Segal, 1996) and even more so if the infants are prematurely born (e.g., Otila, 1948; Bauer et al., 1965). Results from these and other studies are summarized in Table 2. The mean values reported for healthy term babies were in the range 63–115 mg/100 ml. For pre-term infant the mean values in different series were generally higher (up to nearly 190 mg/100 ml). Little information about human fetal CSF is known but Adinolfi published important studies on CSF and plasma proteins obtained from aborted human fetuses (Adinolfi et al., 1976; Adinolfi and Haddad, 1977; Adinolfi, 1985). These studies showed that the concentration of total protein and of individual proteins such as a-fetoprotein, transferrin and IgG were much higher than reported for live-born infants; the highest individual total protein value reported was 730 mg/100 ml at 24 weeks gestation (Adinolfi et al., 1976) although it needs to be born in mind that the CSF samples were obtained from aborted fetuses of uncertain physiological state.

**Table 2:** **Total protein concentration (mg/100 ml) in CSF of full term and pre-term infants estimated during the first few days of life**.

**Authors**	**Mean (mg/100 ml)**	**S.E.M**	**Range**	***n***	**Condition**
**FULL TERM**					
Spiegel-Adolf et al., 1954	103	9.9	–	14	Normal
Widell, 1958	80.9	6.2	–	11	Healthy
Nasralla et al., 1958	115	5.8	46–194	34	Normal
Naidoo, 1968	63	1.6	32–240	135	Healthy
Piliero and Lending, 1959	70	6.0	25–90	35	Normal
Watson, 1964	77	5.3	26–180	51	Cerebral anoxia
Heine et al., 1981^a^	73.0	17.6	53–95	10	No cereb meningitis
Ahmed et al., 1996	80.8	30.8^*^	–	17	Prev. healthy
Chadwick et al., 2011	106	–	94–115^***^	54	No meningitis
Srinivasan et al., 2012	78^^^	137^**^	60–100	130	Normal
**PRE TERM**					
Otila, 1948^b^	100	5.5	50–138	19	Healthy
Nasralla et al., 1958^c^	167	6.4	81–259	49	Normal
Gyllenswärd and Malmström, 1962^d^	176	26	57–292	9	Healthy
Bauer et al., 1965^e^	187	28	30–1600	70	Various
Bartolozzi et al., 1967^f^	62	4.3	12–144	59	Normal
Cole et al., 1974^g^	120	10	–	9	Normal
Sarff et al., 1976	90	–	20–170		No meningitis
Statz and Felgenhauer, 1983^h^	139	77^*^	68–240	10	No pathology
Mhanna et al., 2008^i^	180^^^	–	124–270	10	Suspect sepsis
Srinivasan et al., 2012^j^	104^^^	203^**^	79–131	148	Normal

a *0–4 weeks. No data on birth weights or gestational age*.

b *Birth weights 920-2150 g, Table 19, p. 91*.

c *Birth weights ≤ 4.5 lbs, Table 2, p. 1404*.

d *Birth weights ≤ 2000 g, Table 5, p. 60*.

e *Birth weights 800–2620 g, Figure 1, p. 1018*.

f *Birth weights ≤ 2500 g, Figure 2, p. 299*.

g *Birth weights 1080–1710 g, Table 1, p. 724*.

h *27–32 weeks, Table 3, p. 158*.

i *27 weeks, values less at younger and older ages*.

j *28–35 weeks, Tables II, p730 and III, p731*.

* *S.D*.

** *95th percentile*.

*** *CIs*.

^ *Median*.

Similar results, however, have been obtained for numerous animal species where it was possible to obtain CSF and plasma samples from a wide range of stages of fetal and neonatal development collected under good physiological conditions. The results from animal studies have been reviewed by Saunders (1977, 1992) and by Dziegielewska and Saunders (1988). One of the earliest studies was by Klosovskii (1963) in cat embryos in which CSF protein concentration was up to 22 times that in the adult. Klosovskii correctly interpreted this as having some nutritive function in the developing brain rather than ascribing it to barrier immaturity. The concentration of total protein and of individual proteins in fetal CSF is much higher than in the newborn and adult, but if samples were obtained early enough in development the level was lower than a peak that occurred in all species probably coinciding with the period of maximum neurogenesis. Although Adinolfi (1985) and others interpreted the high CSF concentration of protein in CSF in the developing brain as evidence of a leaky blood-brain and/or blood-CSF barrier there is now substantial evidence that it is a consequence of specific transport of proteins from blood to CSF via a subpopulation of choroid plexus epithelial cells (Dziegielewska et al., 1980, 1991; Habgood et al., 1992; Knott et al., 1997; Johansson et al., 2006, 2008; Liddelow et al., 2009, 2011a,b). In addition it should be noted that the concentration of protein in any space is a function of its amounts and volume in which they distribute. As has been shown by Johansson et al. (2008) the *amount* of protein in the adult CSF is actually greater in the fetus due to a combination of expanding of the ventricular system and increased CSF flow.

## Induction of tight junctions in brain barrier interfaces

Another widespread misconception is that astrocytes are essential for the induction of tight junctions in cerebral blood vessels in the developing brain. In adult brain end feet of astrocytes encircle cerebral capillaries (Caley and Maxwell, 1970; Xu and Ling, 1994). They are thought to make significant contributions to blood-brain barrier functions (Abbott et al., 2006). The belief that astrocytes may be involved in tight junction formation in the developing brain seems to stem largely from *in vitro* experiments. In culture the presence of either astrocytes or conditioned medium from cultured astrocytes produced cells with more complex tight junctions (Tao-Cheng et al., 1987) and was essential for the preparation of cerebral endothelial monolayers with high transendothelial resistance (Dehouck et al., 1990; Rubin et al., 1991). Janzer and Raff (1987) are frequently cited as supporting the notion that astrocytes are essential for tight junction formation *in vivo*. However, Holash et al. (1993) showed their results were an artifact (see Saunders et al., 2012, for further discussion). It seems to have been overlooked that there are no astrocytes present in the developing brain when it is first vascularized (Caley and Maxwell, 1970; Daneman et al., 2010a). As first shown by Stewart and Wiley (1981) using chick-quail chimeras, tight junction formation in cerebral vessels is induced by some factor in the neural tissue of the developing brain. The vessels are tight to proteins and small molecules from as early as vessels first grow into the neural tissue (Bauer et al., 1993, 1995; Ek et al., 2006). It is not yet clear what the induction factor(s) is/are. However it seems that the pericytes make an important contribution to tight junction formation (Daneman et al., 2010b). The main period of differentiation of astrocytes and their association with capillaries occur in rodents in the first 3 weeks of postnatal life (Caley and Maxwell, 1970). It may be that astrocytes contribute to tight junction-induction during this period of intense vascularization, and subsequent maintenance of tight junctions, but they are not involved in earlier stages of vascularization of the brain.

## Why is it important to understand functioning of barrier mechanisms in the developing brain?

It seems unsatisfactory that this belief in the “immaturity” or leakiness of the blood-brain barrier has persisted into the 21st Century in spite of much evidence to the contrary as reviewed above. There are two quite different reasons why it is important to understand the functional status of barrier mechanisms in the developing brain. The first is that they are likely to be important for different features of brain development at different stages in its development. This is suggested for example, by changes in gene expression for different efflux and influx mechanisms in both the cerebral vasculature and choroid plexuses during brain development (Virgintino et al., 2000, 2007; Daneman et al., 2010a; Liddelow et al., 2012; Kratzer et al., 2013).

The other reason is that it is clinically important. At present because of a lack of information on the extent to which drugs enter the developing brain of the embryo following administration to the mother, the general advice given by doctors to pregnant women is to avoid taking drugs. However, this is not very helpful advice in the case of patients with severe medical conditions requiring continued treatment such as epilepsy (Forsberg and Wide, 2011) depression and anxiety (El Marroun et al., 2014; Ornoy and Koren, 2014) psychosis (Galbally et al., 2014) cancer (van Hasselt et al., 2014), or severe hypertension (Brown and Garovic, 2014).

In the US it has been estimated that between 30 and 35% of women have taken psychoactive drugs during pregnancy (Goldberg and Nissim, 1994). In a study of nearly 100,000 pregnancies, Andrade et al. (2004) found that 64% of pregnant women received a drug prescription. In a four nation study in Europe (De Vigan et al., 1999) an average of 36% of women interviewed used at least one drug during the first trimester of pregnancy. There was substantial national variation (22.5% in Glasgow and 50 and 40% in two centers in France). Because drugs are not systematically investigated for possible deleterious effects on the developing brain the clinical management of pregnant women may be a difficult decision between what is best for the mother and limiting potential damage to the developing fetal brain. This lack of knowledge not only makes it difficult for doctors to make rational decisions about when to and when not to medicate, but commonly results in non-compliance to prescribed medication, terminating medication or self-medication, as well as alternative natural remedies being used by mothers (Wood et al., 2014). The only information available to clinicians at present consists of collections of reports of adverse effects or lack of such effects in compendia such as Yaffe's index (Briggs et al., 2008) or national data organizations such as the US Food and Drug Administration (FDA) or the Australian Drug Evaluation Committee in pregnancy, which classify drugs into categories A/B/C/D/X, where A are drugs shown to be safe for the human fetus and X known to induce birth defects (see Teratology Society Public Affairs Committee, 1994). The majority of drugs fall into categories B and C, drugs for which there are not enough data in humans to know whether they are safe for the fetus. The consequences of the currently inadequate state of knowledge about the safety of medications administered during pregnancy have been highlighted by Kennedy (2011). This classification has been under sustained criticism from the Teratology Society in the US (Teratology Society Public Affairs Committee, 1994, 2007) primarily on the grounds that the lack of knowledge about drugs used in pregnancy generates difficulties in making clinical decisions and causing considerable anxiety in patients. This classification is now being abandoned in the US, in favor of a new pregnancy and lactation-labeling rule designed to improve risk versus benefit assessment of drugs used in pregnant and nursing mothers (Ramoz and Patel-Shori, 2014).

Drug use during pregnancy is likely to increase in the future due to higher average ages of mothers, increasing the likelihood of pregnancy complications as well as a higher incidence of premature births with significant medical interventions as a consequence. There is also a growing apprehension that environmental toxins may affect the developing brain. One review listed over 200 industrial compounds known to be neurotoxic in humans and experimental studies have shown over 1000 substances (Grandjean and Landrigan, 2006); this list probably only represents a very small fraction of all chemicals that are neurotoxic. It is particularly inappropriate that public bodies such as the Agency for Toxic Substances and Disease registry (ATSDR) at the Centre for Disease Control in the US should perpetuate statements about immaturity of the blood-brain barrier based on out of date information and mythology. This has been discussed in detail by Ek et al. (2012) but this discussion appears to have had no impact on current reports published by the ATSDR. A more hopeful sign is that the CDC has recently convened meetings to discuss developing a more systematic approach to safer medication use in pregnancy (Broussard et al., 2014).

Recent gene expression studies in developing animal brains (Daneman et al., 2010a; Liddelow et al., 2012, submitted; Kratzer et al., 2013) suggest that many of the protective efflux mechanism that prevent entry of drugs and toxins into the adult brain are present and in some cases more highly expressed in blood vessels and choroid plexuses of the developing brain. However, expression does not necessarily equate with functional effectiveness, so there is an urgent need for systematic drug studies in pregnant and newborn animals and people, in order to define clearly the potential hazards or lack of hazards, when drugs are administered to pregnant women.

## Summary

In conclusion, it seems that many of the most strongly held beliefs about the early history of the blood-brain barrier are incorrect. The blood-brain barrier was not described first by Ehrlich (1885); indeed he did not believe that there were any differences in permeability of cerebral blood vessels compared to the rest of the body. Lewandowsky (1900) did not coin the term blood-brain barrier (“Blut-Hirnschranke”). The first person to use this term seems to have been Stern (Stern and Gautier, 1921). Goldmann (1909) was not the first person to demonstrate that some dyes (in his experiments trypan blue) do not enter the brain following parenteral injection. This appears to have been first reported by Franke (1905) who used trypan red. Behnsen (1927) although frequently cited as providing evidence for immaturity of the blood-brain barrier did not do so. Other claims of barrier immaturity or leakiness in developing animals in dye injection experiments can be explained by use of excessive volumes or concentrations of material injected into fragile animals. Early induction of tight junctions in cerebral vessels does not involve astrocytes. At least in the choroid plexus the route of transfer from blood to CSF is intracellular and not via the paracellular pathway.

The importance of discarding the notion of blood-brain barrier immaturity or leakiness in the developing brain lies in the fact that this belief has impeded research into these mechanisms in brain development and their possible relevance for normal and abnormal brain development, as well as the need for systematic studies of therapeutic agents used to treat pregnant women. Therapeutic advice to such patients should be based on evidence, not fear of the unknown.

## Author contributions

All of the listed authors contributed to the conception, design, research, drafting and final approval of the work. They each agree to be accountable for all aspects of the work.

### Conflict of interest statement

The authors declare that the research was conducted in the absence of any commercial or financial relationships that could be construed as a potential conflict of interest.

## Footnotes

^1^Where possible we have worked from original references. Where these were not in English we have given citations of key points in the original language, together with a translation (indicated by italics). Readers will be able to decide for themselves if the translations are accurate and if our deductions from the citations are appropriate.

## Abbreviations

CNS: central nervous system
CSF: cerebrospinal fluid
HRP: horseradish peroxidase.
